# Epoxy toughening through high pressure and shear rate preprocessing

**DOI:** 10.1038/s41598-019-53881-0

**Published:** 2019-11-22

**Authors:** G. Fernández Zapico, Naoto Ohtake, Hiroki Akasaka, J. M. Munoz-Guijosa

**Affiliations:** 1 Mechanical Engineering Department, E. T. S. Ingenieros Industriales, Universidad Politécnica de Madrid. C/ José Gutiérrez Abascal, 2, 28006 Madrid, Spain; 2 Department of Mechanical Engineering, Tokyo Institute of Technology, 2-12-1-I3, O-Okayama, Meguro-ku, Tokyo, 152-8552 Japan

**Keywords:** Mechanical properties, Mechanical engineering

## Abstract

We have successfully conceived and demonstrated a simple, scalable process for improving the fracture energy of epoxy resins. The process is based on the combined application of high pressures (in the order of GPa) and shear rates (in the order of 10^6^ s^−1^) in the pre-cured polymer, obtaining mechanical forces sufficiently high to increase the reactivity of the monomers due to the scission of the epoxy groups. To achieve these high values of pressure and shear rate, we take advantage of the elastohydrodynamic phenomena occurring at lubricated higher kinematic pairs as, for example, the rolling element – track pair in ball bearings. Experimental results show that, under certain combinations of pressure and shear rate, a substantial improvement in fracture toughness is obtained. SEM observations, Raman spectroscopies, nanoindentation and GPC and NMR measurements show that the process is able to increase the polymer chain length before curing, reducing the number of potential anchor points during the subsequent curing and hence reducing the crosslinking density. The chain lengths obtained are big enough to guarantee adequate stiffness and strength due to increased chain tangling, hence overcoming the drawbacks associated with other toughness promotion methods, such as stiffness and strength reduction.

## Introduction

Among thermosetting polymers, epoxy resins are one of the most massively used in applications where a superior mechanical, adhesive and chemical resistance is needed^1,2^. Epoxies are commonly used in various engineering fields, such as coating, structural components, electronic materials, adhesives, composites and laminates among others^3^. However, in terms of their mechanical properties, epoxy resins show brittleness and crack sensitivity^4^. This is one of their major drawbacks, as toughness is one of the most valuable properties demanded of materials in mechanical applications due to its relationship with reliability and safety. Considerable research activity is related to the enhancement of the fracture toughness of epoxies^5,6^.

There are different types of epoxies, those based on the glycidyl ether group being the most common. In this group, Bisphenol A, Bisphenol F and epoxidized Pheno Novolac are widely used, the former being the most popular^4^. The main chemical characteristic of epoxy resins is the presence of epoxide groups, which are usually located at the terminations of the polymer chains. The C-O covalent bonds of the epoxide group form the so-called epoxy ring. This epoxide group is weaker than the other ether groups due to the strain inherent in the three-membered ring^7^; this is consequently a highly desirable point in terms of crosslinking and chain extension during the polymerization process^8^.

Whereas long chain lengths and crosslinking densities lead to high stiffnesses and strengths, high crosslinking densities lead to poor fracture toughness due to the reduced chain motion potential. Consequently, control of epoxy ring stability during curing is a key factor for achieving an optimum mix between chain length and crosslinking density from the number of epoxy rings available. This is a difficult task as epoxide terminations are easily reactive and work as connection points for both chain extension and crosslink. Different strategies have shown effectiveness on the toughness modification in epoxies. Resin formulators and researchers make a constant, substantial effort to develop processes that lead to increased monomer lengths and a reduced degree of functionality, so that longer, less crosslinked chains result after curing^9^. In this case, stiffness and strength are provided at design stresses by chain entanglement rather than by crosslinking but, simultaneously, an increase in toughness is obtained due to the bigger chain mobility at high stresses. Unfortunately, the polymer chain lengths industrially obtainable are limited by the cost of functionalization. Other common toughening methods are elastomer modification, filler addition and thermoplastic modification^4^. In elastomer modification, elastomeric materials are added such as poly(siloxane)s^10^ or acrylated elastomers^11^ among others. Elastomers support the increase in fracture toughness while at the same time reducing Young’s modulus and yield strength. One of the most common polymers found in the literature is carboxyl-terminated butadiene acrylonitrile (CTBN), which highly increases the impact resistance of epoxy resins^12^. Nanofillers have attracted considerable attention in terms of improving the mechanical, thermal, electrical and electronic properties of polymers^13,14^. Graphene, CNTs, nanofibers, nanocellulose, carbon nanoparticles and nanoclays are widely used examples. Considerable increases in stiffness, strength, fracture toughness and fatigue life have been reported when adding small filler contents to epoxies^15–18^. Thermoplastic modification methods use thermoplastic materials such as polyethersulphone^19^ and polyetherimide^20–24^ in a blend with the thermoset material. The main advantage is that the neat epoxy does not suffer decreases in properties such as Young’s modulus or yield strength; however, the toughness increase is poor in comparison to the two last-mentioned methods.

**Figure 1:**
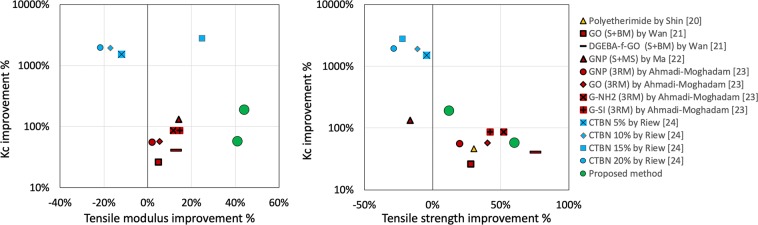
Comparison of the performance of different toughening strategies. Green circles: proposed method.

Figure 1 shows some examples of the different strategies mentioned above. In the Figure, different additives are contemplated as graphene oxide (GO), functionalized graphene oxide (DGEBA-f-GO), graphene nanoplatelets (GNP), amino functionalized GNP (G-NH2), silane modified GNPs (G-Si), and carboxyl terminated butadiene acrylonitrile (CTBN). In addition, different methods are used such as sonication (S), ball milling (BM), mechanical stirring (MS) and three roll mill (3RM).

The main drawbacks of the aforementioned modification treatments are the difficulty of achieving the required conditions for correct polymerization^25,26^, the loss of other properties when toughening the material (especially on elastomer modification), such as flexural modulus and yield strength^27^, and the increased cost of the polymer, especially when using certain nanofillers^4^.

In this work, we propose a novel approach to toughen epoxy resins, in which a combination of high pressures and shear rates is applied to the pre-cured polymer. Mechanical forces affect the inter- and intramolecular bonds due to the distortion of valency angles, increase in interatomic distances or the increase in potential energy produced. This can lead to more reactive bonds due to the higher energy state induced, as well as to scissions, recombinations or the untangling of previously tangled chains^28,29^, which in turn leads to the formation of new functional groups, chain extensions or branching.

Taking advantage of this effect, with the proposed process we create mechanical forces that are high enough to promote the reactivity of the three-membered epoxy rings before curing. This causes longer monomer chains, and consequently to a reduced number of anchor points during curing, leading to a reduced crosslink density. Scissions in the polymer chains are also created, which serve as anchor points for subsequent crosslinking during the curing process, allowing the chain length/crosslinking density ratio to be fine-tuned by means of different combinations of pressure and shear rate.

Experimental results show that, for optimum combinations of pressure and shear rate, a substantial increase in toughness is achieved, with no simultaneous reduction of stiffness or strength. SEM observations show clear differences in the crack surfaces associated to a toughness increase. Gel permeation chromatography (GPC) measurements indicate a clear increase in chain length, while carbon-13 nuclear magnetic resonance (^13^C NMR) and Raman spectroscopy analyses show that the chemical main chain is not modified, consequently validating the hypothesis of increased chain lengths and decreased crosslinking density, which is further demonstrated by creep measurements from nanoindentation tests.

## Results

### Sample preparation

**Figure 2:**
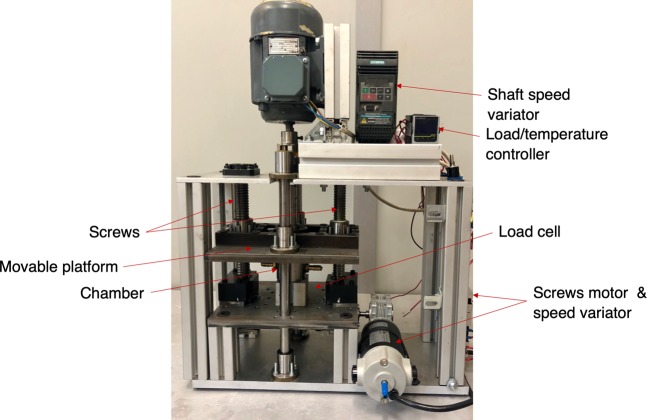
Preprocessing device.

The polymer chosen for this work was Resoltech 1800^30^, an infusion and injection epoxy resin system with low viscosity. It is usually employed for tooling and parts manufacturing. Its high interlaminar shear strength and low viscosity makes it a suitable material for infusion-based processes in carbon fiber laminates. Its monomer is Bisphenol A diglycidyl ether. Resoltech 1805 -Diamine (1.2-diaminocyclohexane)- was used as hardener. Figure 2 shows the machine designed for the exertion of the pressures and shear rates to the pre-cured polymer. An aluminum chamber contains the axial bearing and the pre-cured polymer, in liquid state. A sliding platform, moved by means of two synchronized ball screws, allows for the application of a force up to 5000 kg to the bearing. A shaft is connected to the bearing at one of its ends and to an electric motor. A speed variator allows for the control of that motor and consequently of the bearing speed. A load cell is used to measure the force applied and a thermocouple, submerged in the liquid pre-cured polymer at the chamber, allows for the direct measurement of the pre-cured polymer temperature. The refrigeration process was done by means of a water-cooled copper coil.

Seven different treatment recipes were performed, by application of different combinations of pressures and shear rates to the pre-cured polymer. Temperature was maintained at 60 °C in all cases to avoid temperature-induced degradation and facilitate the process as viscosity is considerably reduced at higher temperatures, leading to a deterioration of the hydrodynamic wedge. The force and speed needed to create the desired pressure and shear rate were calculated by means of the EHD theory (Eqs. 1 to 11), as a function of the resin viscosity and the ball bearing used. As the EHD theory allows for the calculation of the fluid film thickness, the mass flow of material undergoing the desired high pressure and shear rate conditions can be obtained and, consequently, the time needed for the processing of a certain amount of fluid can be calculated. Table 1 shows the sample families obtained and the process conditions undergone by each one. Five specimens were obtained on each sample family for further characterization.

**Table 1:** Process parameters applied to the different sample families.

Sample Family	Pressure (GPa)	Shear rate (×10^6^ s^-1^)
A	0	0
B	0.2	1.34
C	0.2	2.96
D	1	2.14
E	1	4.71
F	1.5	2.35
G	1.5	5.12

It is important to highlight that pressure and shear rate cannot be totally decoupled, as zero shear rate would imply the absence of a hydrodynamic wedge and, consequently, would produce a ball-track contact. Similarly, zero pressure would lead to a theoretically infinite wedge, impossible to achieve in practice. Furthermore, the absence of axial load in the bearing would lead to substantial vibrations in the system. Nevertheless, in order to try to reveal the separate effect of pressure and shear rate, we have prepared sample families B and C with very low pressure.

Subsequently, hardener was mixed for 10 minutes. Afterwards, resin was degassed in a vacuum chamber for 15 minutes and finally injected in the corresponding silicon molds, with the cavity geometry required for the creation of the tensile and fracture test specimens. The curing process was conducted according to the supplier specifications: 16 hours in a conventional oven at 60 °C.

### Mechanical performance

**Figure 3:**
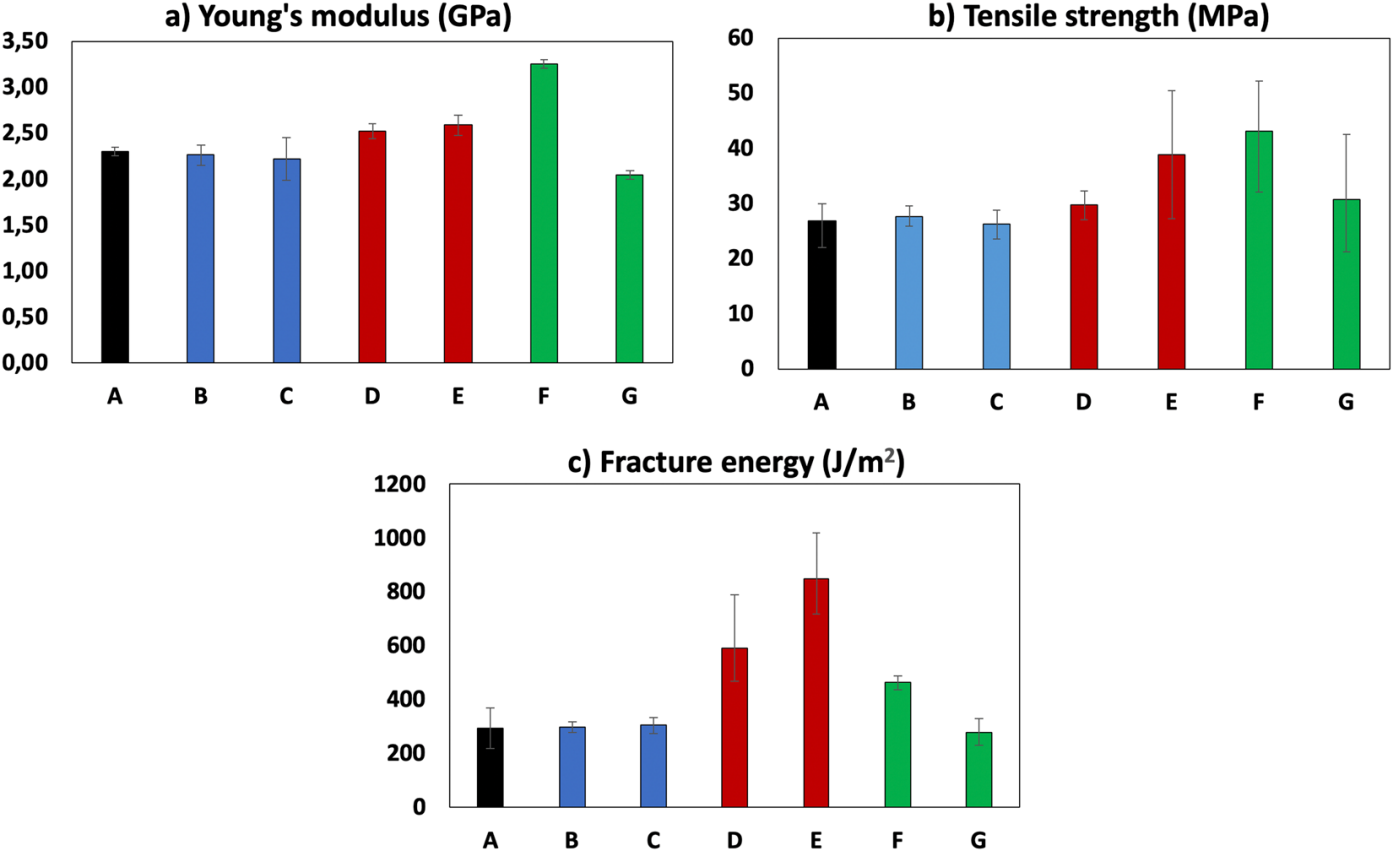
Mechanical tests results.

Figure 3a,b,c show the results of the Young’s modulus, tensile strength and fracture energy tests, respectively. While no substantial changes are detected in the samples which were treated with very low pressure (families B and C), substantial variations were found in the samples which undercame treatments with simultaneous pressure and shear rate application: regarding fracture energy, sample family D shows an increase of 110% and family E of 188%. Regarding tensile strength and Young’s modulus, maximum increases of +62% and +41% were found, respectively, on the sample family F. These figures allow for a remarkably advantageous toughening method with respect to the aforementioned ones, as depicted in Fig. 1.

### Structure and morphology

**Figure 4:**
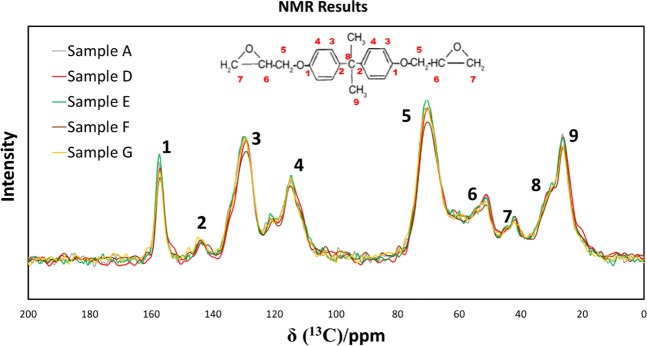
NMR spectra.

**Figure 5:**
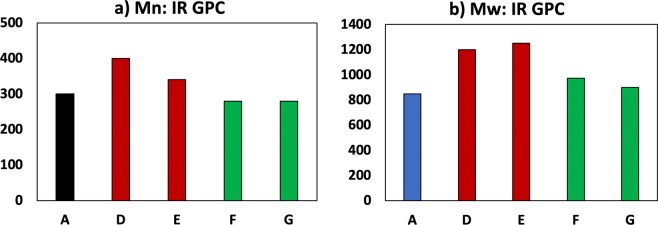
GPC results.

Figure 4 depicts the NMR spectra performed on the sample families which showed modifications in the mechanical properties. Although very small shifts are observed in the representative peaks, no differences in the polymer structure were found. Raman spectra show accordance with the NMR spectra, as it is not possible to appreciate big peak shifts between the samples. The results of the GPC analyses on the same samples are shown in Fig. 5a,b. *M*_*n*_ represents the number average molecular number and *M*_*w*_ the weight average molecular weight. Both figures show how 1 GPa pressure increased the *M*_n_ up to 25%, while *M*_w_ is increased up to 50% with respect to the non-treated samples. A clear correlation is found between the values of the tensile strength, stiffness and fracture energy and the GPC values is found.

**Figure 6:**
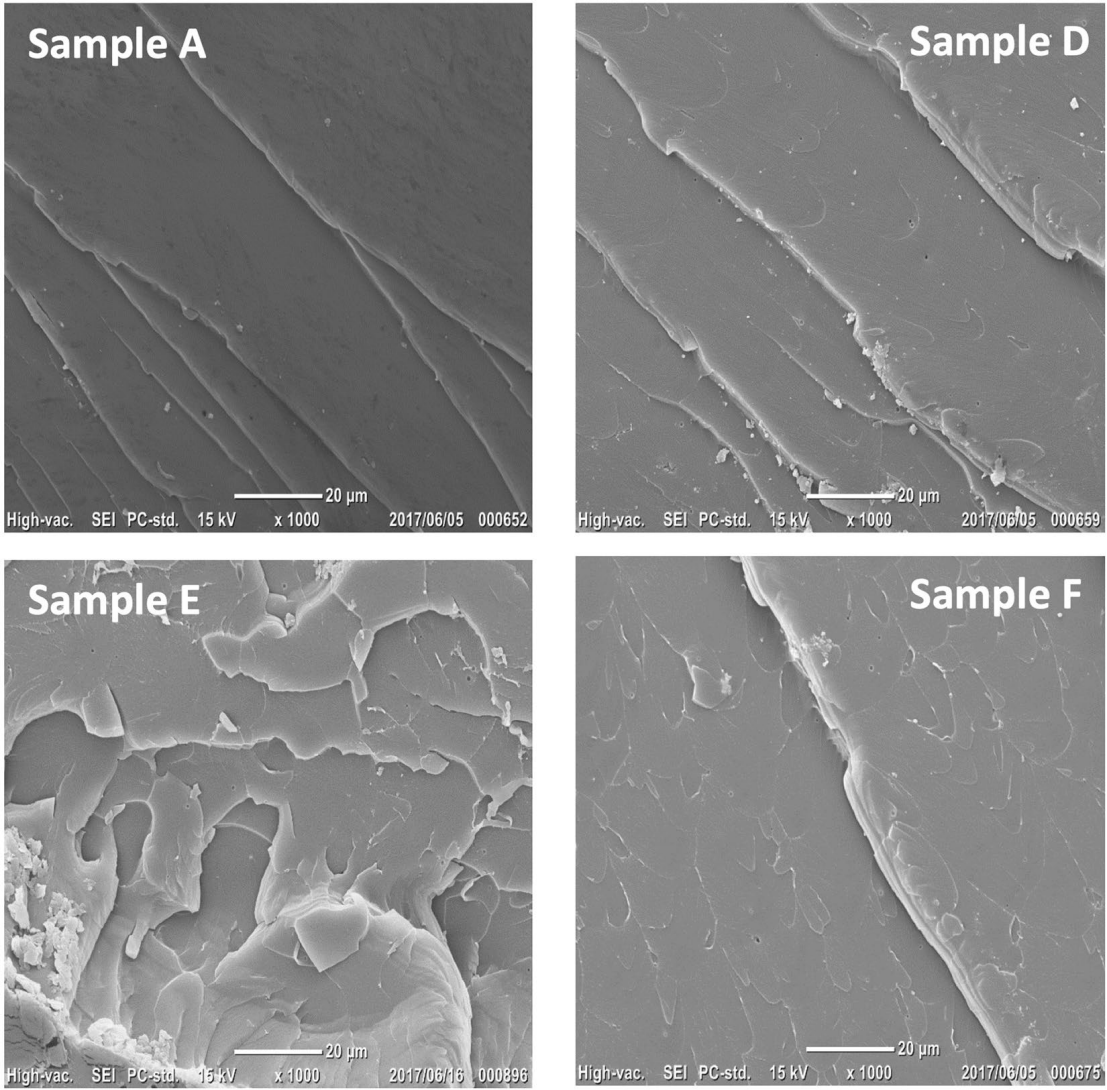
SEM observations: Effect, with respect to the non-processed material (sample A) of an increase in applied pressure and shear rate (sample D), an increase in shear rate (Sample E) and an increase in pressure (sample F).

In addition to the mechanical tests, SEM was used to analyze the morphology of the crack surfaces in the same sample families. The effect of the pressure and shear rate is shown in Fig. 6, by comparison, with respect to the non-preprocessed material (sample A), of an increase in pressure and shear rate (sample A to sample D), an increase in shear rate with constant pressure (sample D to sample E) and an increase in pressure with constant shear rate (sample E to sample F). A pressure and shear rate increase leads to a clearly rougher surface and slightly different crack propagation directions (sample D). A higher shear rate leads to an even rougher crack surface, presenting circular marks and some scratches in different directions, whereas a higher pressure also leads to a bigger crack surface extension. All the above characteristics are coherent with the modifications in fracture toughness measured.

**Figure 7:**
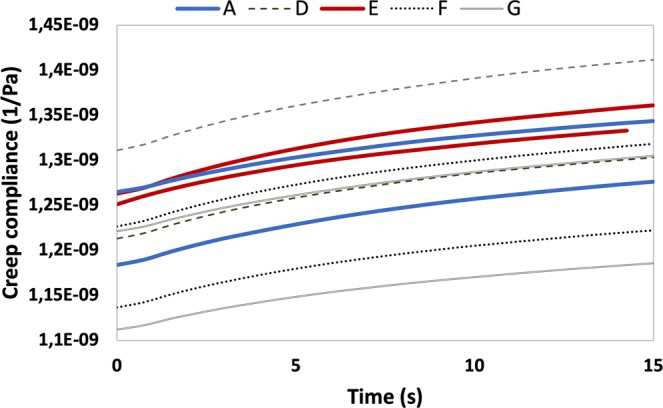
Creep compliance evolution with time. Continuous red and blue lines represent the results of the base case (not processed epoxy) and the sample family which showed maximum fracture energy increase, respectively.

Indentation tests were also carried out in order to check the creep compliance. Creep compliance is a good indicator of molecular weight between crosslinks and hence crosslinking density^31,32^. Results for each sample family are shown in Fig. 7. An increase in creep compliance is observed in samples D and E, followed by a reduction in samples F and G, which lead to compliance values even smaller than those measured in the base case sample family (not processed epoxy). Creep compliance rate appears nearly invariable in all the samples.

## Discussion

From the NMR results, it may be concluded that the polymer chain structure has not undergone changes due to the process. Raman results, which do not indicate any substantial difference in the spectra of the treated specimens with respect with the not treated ones, are in accordance with NMR, reinforcing the conclusion that no chemical changes on the polymer are caused by the treatment. However, the increases detected in the GPC M_N_ and M_W_ for the samples which were simultaneously processed with both pressure and shear rate reveal longer chains in the treated samples. Pressure and shear rate, and not temperature, seem to be the cause of the substantial changes in the chain length, as temperature was maintained constant in all the processing recipes.

A clear correlation between the fracture energy, tensile strength and stiffness and the *M*_W_ value is found. This can be explained as follows: a pre-cured polymer chain length extension is obtained prior to the curing process due to the polymerization reactions allowed by the opening of the epoxy rings -which are the most reactive specimens present-^33^ produced by the pressure and shear applied. This consequently reduces the number of potential anchor points during the curing, leading to a reduced crosslinking density. These phenomena do not necessarily lead to a reduction in stiffness or strength in the final polymer due to the increased chain tangling associated to the bigger chain lengths. However, the mechanical forces provided with the process simultaneously induce a competitive mechanism of chain scission, especially in the longest chains, as demonstrated by^29,34^. This gives rise to potential anchor points for crosslinking during the curing.

For low pressure and shear rate values (sample D), the effect of chain extension seems to be predominant, leading to longer chains and slightly reduced crosslinking density, as the increased GPC Mw and creep compliance show, respectively, resulting in an increase in both stiffness, strength and fracture energy. An increased shear rate holding the same pressure (sample E) leads to a slight decrease of the extension/scission ratio due to the increased energy introduced, with no appreciable change in the length of the longer chains. This is coherent with the increase in the *M*_w_/*M*_N_ ratio found in sample E with respect to sample D, which suggests a wider chain length distribution. Crosslinking density seems not modified according to the creep compliance measured. This particular combination of chain length and crosslinking density seems to be optimum for chain entanglement and mobility and, consequently, for fracture energy. The constancy of creep compliance rate is actually other evidence of crosslinking density reduction, as usually reductions are expected for higher chain lengths^32^.

For excessive pressures (samples F and G), the scission effect seems to be predominant, leading to higher degrees of crosslinking, which result in higher stiffness and strength (sample F), but also an increased brittleness as a result. Finally, if shear rate is also increased (sample G), the excessive scission reduces the chain lengths so much that, even with increased crosslinking density, a reduction in all the mechanical parameters is observed. The mechanical energy introduced in samples B and C, where a practically null pressure was applied, seems not to be enough to induce the aforementioned mechanisms, according to the almost inappreciable effect observed.

We believe the proposed method can be advantageous in other applications requiring high shear rate, as dispersion, mixing or the avoidance of particle reaggregation.

## Methods

### Elastohydrodynamic theory in the contacts occurring in lubricated, superior kinematic pairs

Higher kinematic pairs are those characterized by the presence of a theoretically linear or punctual contact between its components, as well as a relative sliding, rolling and/or pivoting speed at that contact. Examples of higher kinematic pairs are the rolling element-track pair in rolling bearings or the interaction of two teeth in meshed gearwheels. Very high pressures (in the order of GPa) can arise in the contact between each element of the pair, due to the very small contact surface produced by the deformation of the elements, which in practice can be observed as an ellipse (with axes whose length is in the order of 10^−6^m) or a rectangle (where the short edge is in the same order of magnitude). These small values are a result of the geometries used for each element of the pair and the high stiffness of the materials employed -metals or ceramics-, which are frequently subjected to surface hardness treatments. The resulting contact stress distributions are very complex, and can be calculated by means of the Hertz contact theory^35^. In correctly lubricated higher kinematic pairs, no contact between each element of the pair exists, due to the formation of a lubricant film between them. If a sufficiently high η*v*/*p* exists, η being the lubricant dynamic viscosity, *v* the relative speed and *p* the existing lubricant pressure, no external pressure is needed to create this fluid film, as a hydrodynamic wedge appears, where pressure is high enough to overcome the contact force and separate both members of the pair. The calculation of the wedge thickness and the resulting stress distributions involves the coupled use of Hertz theory and fluid dynamics equations. This strategy is usually known as “Elastohydrodynamic lubrication theory” (EHD)^36^. The EHD theory shows that, despite the absence of contact, the deformation of the members leads to footprint surfaces at each member which are very similar, in shape and extension, to those occurring in non-lubricated contacts. The pressures to which the fluid and the pair members are exposed are also very similar to those occurring at the contact when no lubrication exists. For theoretically punctual contacts, such as those arising between the ball and the tracks in a thrust ball bearing, the resulting footprint has an elliptical shape. The contact pressure can be calculated by means of (1), as a function of the length of the ellipse axes and the force applied *F*_*esf*_, which in turn can be calculated as the fraction of the total force *F* applied on the bearing and the number *z* of rolling elements.

The extension of the elliptical footprint will strongly depend on the force applied *F*_*esf*_, as well as on the curvature radii of the ball and the tracks, expressed by an equivalent radius *R*_*eq*_, which considers the possible differences in the curvatures *R*_*x*_ and *R*_*y*_ along the main axes of the ball (*R*_*X1*_ and *R*_*Y1*_) and the track (*R*_*X2*_ and *R*_*Y2*_); the equivalent Young’s Modulus (*E*’) that depends on the Young’s modulus of each member and its Poisson’s ratio; and specific parameters *a*_1_ and *b*_*1*_ which are dependent on the ellipticity of the footprint.

1 $${p}_{0}=\frac{{F}_{esf}}{\pi ab}=\frac{F/z}{\pi ab}$$

2 $$a={a}_{1}{(3{F}_{esf}\frac{{R}_{eq}}{E^{\prime} })}^{1/3}$$

3 $$b={b}_{1}{(3{F}_{esf}\frac{{R}_{eq}}{E^{\prime} })}^{1/3}$$

4 $$\frac{1}{{R}_{eq}}=(\frac{1}{{R}_{X}}+\frac{1}{{R}_{Y}})$$

5 $$\frac{1}{{R}_{X}}=(\frac{1}{{R}_{1X}}+\frac{1}{{R}_{2X}}),\frac{1}{{R}_{Y}}=(\frac{1}{{R}_{1Y}}+\frac{1}{{R}_{2Y}})$$

6 $$\frac{2}{E^{\prime} }=[\frac{1-{v}_{1}^{2}}{{E}_{1}}+\frac{1-{v}_{2}^{2}}{{E}_{2}}]$$

7 $${a}_{1}=k{(1+\frac{2(1-{k}^{2})}{\pi {k}^{2}}-0.25\log (k))}^{1/3}$$

8 $${b}_{1}=\frac{{a}_{1}}{k}$$

9 $$k={(1+\sqrt{\frac{\log (16/D)}{2D}}-\sqrt{\log 4}+0.16\log D)}^{-1}{\rm{where}}\,D=\,{\rm{\min }}({R}_{X}{R}_{Y}\,,{R}_{Y}/{R}_{X})$$

The film thickness *h*_*cN*_ can be obtained by different semianalytic models. For instance, Dowson and Hamrock^37^ proposed the expression shown in (10), where α and η are the viscosity-pressure coefficient and the dynamic viscosity of the lubricant, respectively; *E*’ is the equivalent Young’s modulus of the materials (6), *R* the equivalent curvature radius (4), *u*_*m*_ the relative speed at the pair (11) and *p*_*o*_ the contact pressure (1).

10 $${h}_{cN}=1.55{({\alpha }^{\ast }(T))}^{0.53}{({\eta }_{0}(T){u}_{m})}^{0.67}{(E^{\prime} )}^{0.061}{{\rm{R}}}_{Eq}^{0.33}{{p}_{0}}^{-0.201}$$

11 $${u}_{m}=({V}_{Ball}+{V}_{track})$$

### Viscosity measurement

The polymer viscosity was calculated in a Couette viscosimeter for three different temperatures.

### Sample characterization

SEM observations were carried out in a SEM JCM-6000 (JEOL) after a 75-second gold sputtering process. High-resolution solid-state NMR analyses were conducted using a JNM-ECA400 (JEOL) at resonance frequencies of 100 MHz for carbon-13. The chemical shift of ^13^C NMR spectra was determined at room temperatures. GPC measurements were carried out in a JASCO HSS-1500 system with TSK gel α-3000 column (TOSOH) using DMF and LiBr, as solvent. Raman spectroscopies were carried out in a NRS-4100 Laser Raman Spectrometer using laser 532 nm in wavelength. The fracture toughness tests were conducted in an Instron floor machine according to ASTM D5045. The tensile strength and stiffness tests were carried out according to ASTM D638. Creep compliance was measured by means of indentation tests in a Elionix device with a Berkovich diamond tip, by applying a load up to 100 mN at a rate of 2 mN/s, and a holding time at maximum load of 15 seconds.

## Supplementary information

Supplementary information
