# Author Correction: Epoxy toughening through high pressure and shear rate preprocessing

**DOI:** 10.1038/s41598-019-55967-1

**Published:** 2019-12-24

**Authors:** G. Fernández Zapico, Naoto Ohtake, Hiroki Akasaka, J. M. Munoz-Guijosa

**Affiliations:** 1 Mechanical Engineering Department, E. T. S. Ingenieros Industriales, Universidad Politécnica de Madrid. C/José Gutiérrez Abascal, 2, 28006 Madrid, Spain; 2 Department of Mechanical Engineering, Tokyo Institute of Technology, 2-12-1-I3, O-Okayama, Meguro-ku, Tokyo, 152-8552 Japan

Correction to: *Scientific Reports* 10.1038/s41598-019-53881-0, published online 22 November 2019

The original version of this Article contained a typographical error in the Abstract.

“The process is based on the combined application of high pressures (in the order of GPa) and shear rates (in the order of 10^6^ m^−1^) in the pre-cured polymer, obtaining mechanical forces sufficiently high to increase the reactivity of the monomers due to the scission of the epoxy groups”.

now reads:

“The process is based on the combined application of high pressures (in the order of GPa) and shear rates (in the order of 10^6^ s^−1^) in the pre-cured polymer, obtaining mechanical forces sufficiently high to increase the reactivity of the monomers due to the scission of the epoxy groups”.

Additionally, the original version of this Article contained a typographical error in the first line, third column of Table 1.

“Shear rate (×10^6^ m^−1^)”

now reads:

“Shear rate (×10^6^ s^−1^)”

These errors have now been corrected in the PDF and HTML versions of the Article.

