# Supplementary material for: Epoxy toughening through high pressure and shear rate preprocessing
Source: Sci Rep. 2019 Nov 22;9:17343. doi: 10.1038/s41598-019-53881-0 (PMC6874574; doi:10.1038/s41598-019-53881-0)
Supplement: Supplementary file 1 — Supplementary information [file 41598_2019_53881_MOESM1_ESM.pdf]

Epoxy toughening through high pressure and shear rate preprocessing

G. Fernández Zapico[1], Naoto Ohtake[2], Hiroki Akasaka[2], J.M. Munoz-Guijosa[1*]

Supplementary information

S1. Sample Preparation

The ball bearing used for the exertion of pressure and shear rate to the pre-cured polymer was a SKF 52205. After the high pressure and shear rate processing, the pre-cured polymer is on a fluid state, which makes it easy to mix and pour.

Samples were prepared by adding the hardener to the processed pre-cured polymer in a weight ratio of 100/17 as specified by the supplier specifications. Both components are mixed during 10 minutes and afterwards degassed during 15 minutes in a vacuum machine (Figure **S1.1**).

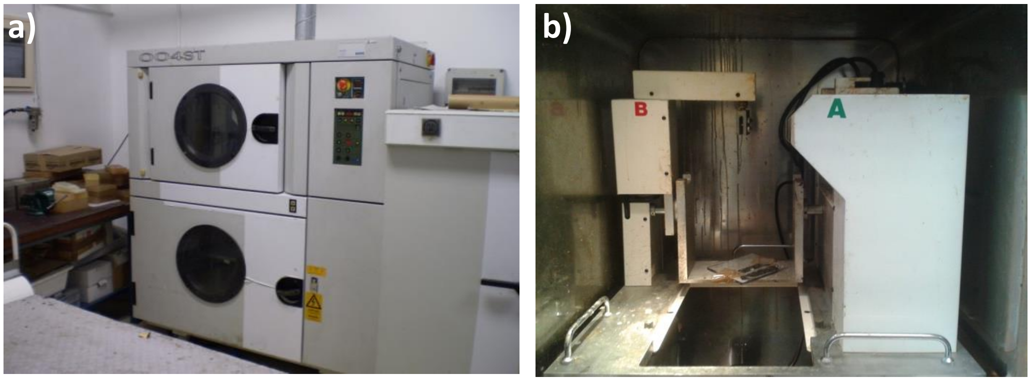

Figure S1.1 a) Vacuum machine. b) Detail of the vacuum machine mixer.

To obtain the required ASTM shapes, silicone moulds (Fig. **S1.2d**) were used, into which the processed, mixed and degassed pre-cured polymer is casted. Two different types of moulds are used, for tensile and fracture tests. Master models (Fig. **S1.2c**) were used to fabricate the moulds. The master models are manufactured by fused deposition 3D printing of PLA. Fig. **S1.2a** shows the design of the fracture test specimen and Fig. **S1.2b** the design of the tensile test specimen master models. Silicon was cured according to the supplier specifications (5 hours at ambient temperature).

Finally, the mix is poured in the moulds, slightly over the mold surface. Curing was performed according to supplier specifications, during 16 hours and 60ºC in oven.

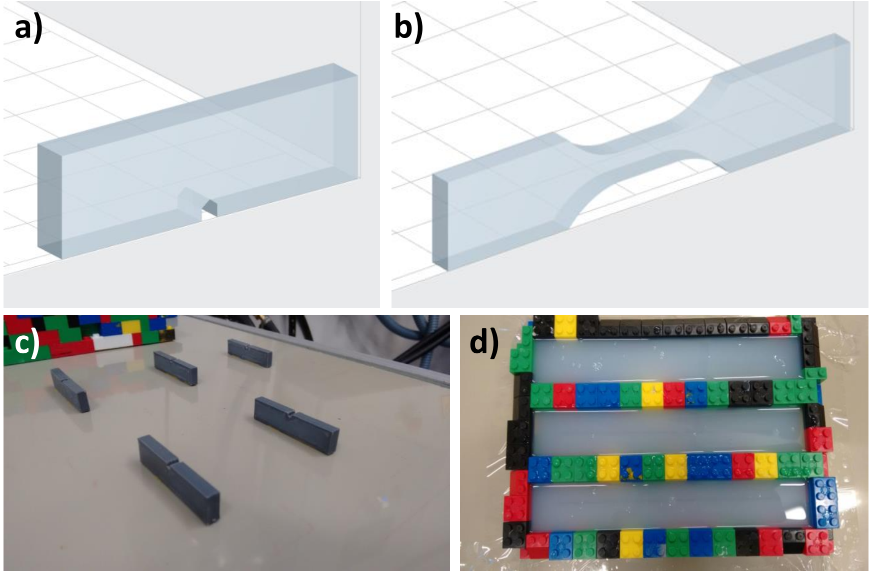

Figure S1.2. a), b) 3D models of the tensile and fracture energy specimens. c) 3D printed models. d) Silicon poured over the 3D printed models.

S2. NMR

Fig. **S2.1**. shows the chemical chain that is studied for the NMR analysis. The different numbers correspond to the peak position.

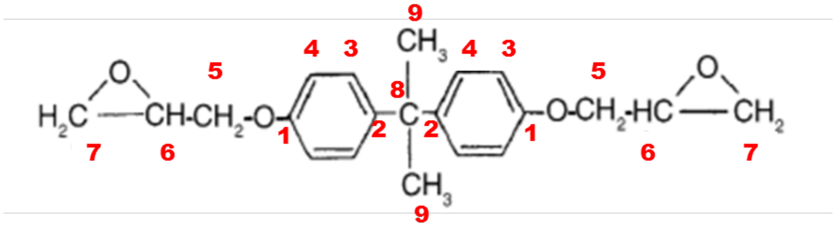

Figure S2.1. Final chemical form from NMR analysis.

The results of non-treated and treated neat epoxy resin are shown in Fig. **S2.2**. X axis represents the parts per million of Carbon 13. Y axis represents the test intensity. Each peak has a number which corresponds to a chemical bond marked in the chemical chain represented in Fig. **S2.1**. For a better results interpretation, a comparison of the measurement spectra of each sample with Sample A (non-treated epoxy) is added.

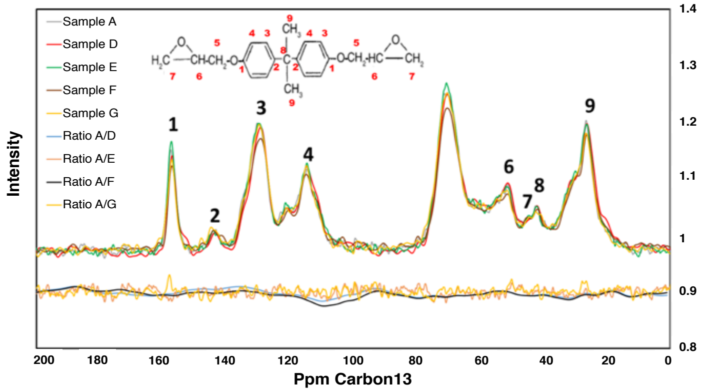

Figure S2.2. Neat epoxy NMR results

S3. RAMAN

Raman spectrography results are shown in Fig. **S3.1.** for neat epoxy. As usual, as intensities are in arbitrary units, they were shifted to better visualize the results. Two main peaks are found in the neat epoxy samples, at 1450 cm^{-1} and 1610 cm^{-1}. As observed in Fig. **S3.2.**, in the 1450 cm^{-1} peak there is a shift to the right but never higher than 0.25%. At 1610 cm^{-1} there is not an appreciable difference between samples.

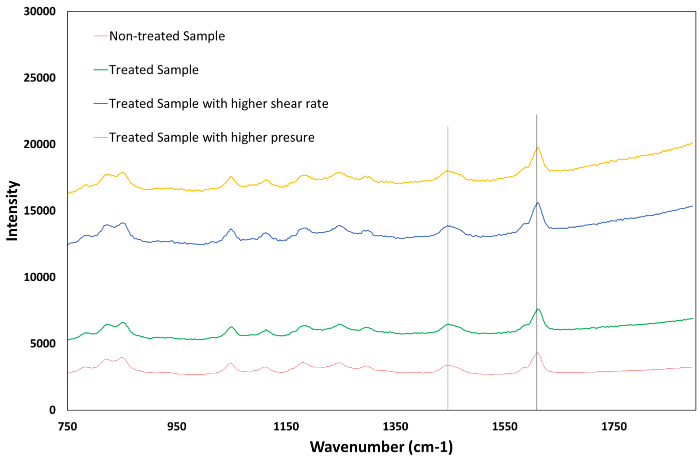

Figure S3.1. Raman results for epoxy specimens

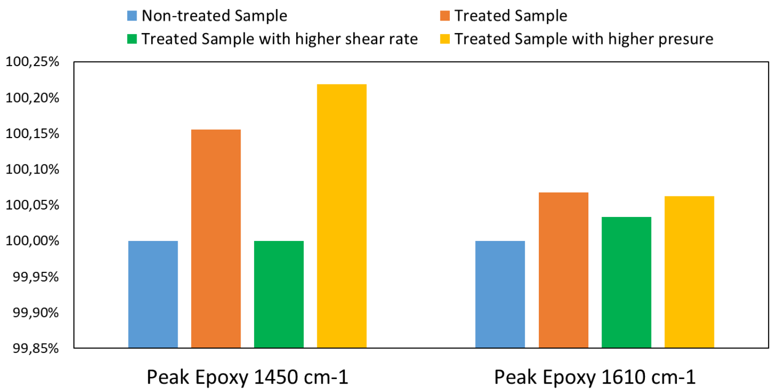

Figure S3.2. Raman results for epoxy specimens. Peak shift analysis.

S4. GPC

The GPC analyses were performed with DMF+LiBr as solvent. Concentration was around 2 g/mL. The column used was a TSKgel α-3000, TOSOH. IR and UV analyses were carried out.

S5. CREEP COMPLIANCE

Creep compliance was calculated by means of Eq. S4.1[28],

$$J_C(t) = \frac{8\tan(\alpha) h^2(t)}{\pi P_0} \quad \text{(S4.1)}$$

Where α is the angle complementary to the angle between the material free surface and the indenter surface, P_0 is the constant load maintained and h the indentation depth. Fig. **S3.3a** and Fig. **S3.3b** show the indentation footprints for samples A and E, respectively.

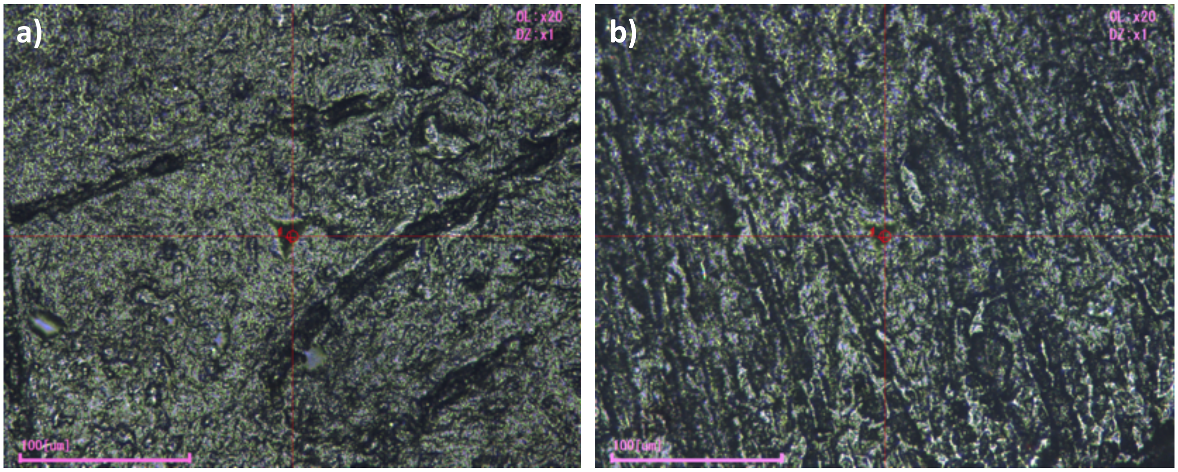

Figure S5.1. Indentation marks in samples A (Figure S3.3a) and B (Figure S3.3b).
